# Influence of lesion size on shear wave elastography in the diagnosis of benign and malignant thyroid nodules

**DOI:** 10.1038/s41598-021-01114-8

**Published:** 2021-11-03

**Authors:** Huizhan Li, Chunsong Kang, Jiping Xue, Liwei Jing, Junwang Miao

**Affiliations:** 1 Department of Ultrasonography, Bethune Hospital Affiliated to Shanxi Medical University, 99 Longcheng Street, Taiyuan City, 030032 Shanxi Province China; 2 Department of Health Statistics, Shanxi Medical University, 56 XinJian South Road, Taiyuan City, 030001 Shanxi Province China

**Keywords:** Cancer, Medical research, Oncology

## Abstract

In shear wave elastography (SWE) studies, the optimal cutoff value of Young’s modulus for the diagnosis of benign and malignant thyroid nodules varies greatly, which affects the clinical application of the method. The objective of this study was to evaluate the influence of thyroid nodule size on the clinical diagnostic efficacy of SWE. A total of 356 thyroid nodules of 280 patients were divided into three groups according to size (Group A: ≤ 1 cm; Group B: 1–2 cm; Group C: ≥ 2 cm). SWE was used to measure the maximum Young's modulus (Emax) values of all thyroid nodules. Receiver operating characteristic (ROC) curves were drawn with pathological results as the gold standard. For all nodules, the optimal cutoff value of Emax in SWE for diagnosing malignant thyroid nodules was 36.2 kPa. The sensitivity and specificity were 76.5% and 78.4%, respectively. Groups A, B, and C had different optimal Emax cutoff values of 33.7 kPa, 37.7 kPa, and 55.1 kPa, respectively. The area under the ROC curve (AUC) values of Groups A, B, and C (0.844, 0.886, and 0.935, respectively) were all greater than the values for all lesions (0.830). The specificity values of Groups A, B, and C (86.4%, 82.6%, and 88.2%, respectively) were all increased, and the sensitivity values of Groups B and C (89.7% and 96.4%, respectively) were also increased compared with the values for all lesions. Thyroid nodule size affects the optimal Emax cutoff value of SWE. We suggest that different cutoff values be used to diagnose benign and malignant thyroid nodules according to lesion size.

## Introduction

Thyroid nodules are a common clinical problem. The detection rate of thyroid nodules using high-resolution ultrasound (US) in randomly selected individuals has been measured as 19–68%, of which 7%–15% are thyroid cancers^1^. Benign and malignant thyroid nodules are treated differently: the 2015 edition of the American Thyroid Association’s “Guidelines for the Diagnosis and Treatment of Adult Thyroid Nodule and Differential Thyroid Cancer” recommends surgery for malignant thyroid nodules but no further treatment for benign nodules^1^. Therefore, it is important to distinguish between benign and malignant thyroid nodules. At present, US is the preferred imaging method for thyroid nodules, but it has limitations in differential diagnosis between atypical benign and malignant nodules.

Thyroid nodule stiffness is closely related to their histopathology^2^. Benign nodules have many follicles and colloidal components, therefore, their stiffness is low. In contrast, malignant nodules contain many fibrous vascular interstitial components and sand-like calcified bodies; thus, their tissue stiffness is relatively high. Further, tissue stiffness can be an important indicator in the differentiation between benign and malignant nodules. Shear wave elastography (SWE) can be used to quantitatively measure tissue stiffness using Young’s modulus values(including Emax, Emean, Emin and Esd which were derived from a region-of-interest analysis), and it is currently widely used for the differential diagnosis of benign and malignant thyroid nodules. The maximum value measured by SWE (Emax) is the most commonly used parameter, and the sensitivity and specificity of SWE for differentiating benign from malignant thyroid nodules are 0.79–0.86 and 0.84–0.90, respectively^3^. However, there is no consensus regarding the diagnostic threshold of Emax, and various cutoff values have been proposed (36.5–94.0 kPa)^4–13^. This situation may be related to the various sizes of thyroid nodules: lesion size has been reported to affect the Emax value^2,7,14,15^. In this study, we stratified thyroid nodules according to their size and assessed the influence of lesion size on the diagnostic efficiency of SWE.

## Materials and methods

### Patients

From January 2017 to June 2019, we enrolled 280 consecutive patients (216 women and 64 men) with 356 thyroid nodules in the study. Their median age was 48 years (range: 24–77 years). The inclusion criteria were as follows: (1) the patients underwent thyroid surgery and had pathological results; (2) the patients had not been previously treated for thyroid nodules; and (3) the patients had no history of radiotherapy of the head and neck regions. The exclusion criteria were as follows: (1) more than 25% of the nodule consisted of the cystic component (because shear waves cannot propagate in liquid); (2) the nodule contained coarse or rim calcifications, which cause information loss in SWE images; (3) the nodule was located in the isthmus or adjacent to the cartilage of the trachea and common carotid artery (because it was difficult to distinguish between actual stiffness and artifacts); and (4) benign and malignant nodules appeared in the same thyroid lobe (because it is difficult to determine the pathological nature of the target nodule).

Of these patients, 217 (77.5%) presented with a single nodule, and 63 (22.5%) exhibited multiple nodules. The size of the 356 thyroid nodules ranged from 0.3 to 3.7 cm. The nodules were divided into three groups based on lesion size (Group A, ≤ 1 cm; Group B, 1–2 cm; and Group C, ≥ 2 cm).

Histology after thyroid surgery was used as the reference method for the diagnosis of malignant thyroid nodules.

### Ultrasonographic examinations

Thyroid B-mode ultrasonography (US) and SWE examinations were performed with an Aixplorer US system (SuperSonic Imagine, Aix-en-Provence, France), which was equipped with an SL15-4 multifrequency linear array transducer. All nodules were examined by the same radiologist who has more than 10 years of experience in the differential diagnosis of thyroid diseases and was proficient in SWE image collection.

#### B-mode US

Before the US examination, patients were placed in the supine position, and the neck was fully exposed. The basic characteristics of the nodules, such as internal structure, echo intensity, boundary, and calcification, were observed using two-dimensional US. Each nodule’s maximum diameter was measured, and the distribution of the nodule’s blood flow signal was observed by color Doppler flow imaging.

#### SWE

After the B-mode US examination, SWE was performed with the same US machine and transducer. The target nodule was identified using two-dimensional US and displayed on the long axis section of the thyroid, and then the image was switched to SWE mode (display Young’s modulus scale: 0–100 kPa). A region of interest including the whole lesion and the surrounding normal thyroid tissue was placed on the nodule, and stiffness was displayed as a color-coded image, with blue and red representing soft and stiff tissue, respectively. The display was frozen when the SWE image was stable. Emax was chosen vs. Emean because the Q-box was round: however, the thyroid nodule was not round,and the Q-box could not cover only the whole thyroid nodule.Emax reflects the stiffest value in the thyroid nodule, while Emean reflects only the average value of the tissue covered by the Q-box, not the average value of the nodule. The Emax value of the nodule was measured using the Q-box for three independent acquisitions, and the mean of the three Emax values was recorded for analysis.

### Statistical analysis

The Emax values of all lesions were correlated with the nodules’ pathological diagnosis. The R software package(R Foundation for Statistical Computing, Vienna, Austria) was used for all statistical analyses in our study. Two-tailed *p* < 0.05 was considered to be statistically significant. Shapiro–Wilk tests were used for distribution normality. Descriptive statistics were expressed as medians (25th and 75th percentiles) or mean values ± standard deviations for continuous data. The Student’s t-test(paired-samples t-test or independent-samples t-test) or Mann–Whitney U test (2 related samples or 2 independent samples) were used to assess the differences between two groups of quantitative variables. The ANOVA or Kruskal–Wallis H tests were used to assess the differences among three groups of quantitative variables. In each group, the diagnostic value of Emax for distinguishing between benign and malignant nodules was assessed by receiver operating characteristic (ROC) curve analysis. The area under the ROC curve (AUC) was calculated, and AUC values were compared using Z tests. Using these curves to establish cutoff points, sensitivity and specificity values were established. For comparisons of sensitivity and specificity between groups, the McNemar test was used.

### Ethics declarations

This study was approved by the Medical Ethical Committee of Bethune Hospital Affiliated to Shanxi Medical University. This research was performed in accordance with relevant guidelines and regulations, and a written informed consent with signature was obtained from all participants or their legal guardians.

## Results

Of the 356 nodules in 280 patients, the pathological findings showed 272 malignant nodules, all of which were papillary thyroid carcinomas, and 84 benign nodules, including 78 nodular goiters and 6 adenomas. The conventional US characteristics of 356 thyroid nodules are presented in Table 1.The Emax values of the 356 thyroid nodules were non-normally distributed.

**Table 1:** Conventional US characteristics of thyroid nodules.

Characteristics	Benign	Malignant
Nodules (n = 356)	84	272
**Maximum diameter**		
≤ 1 cm	44	176
1–2 cm	23	68
≥ 2 cm	17	28
**Location**		
Superior pole	7	56
Middle	53	124
Inferior pole	24	96
**Aspect ratio**		
> 1	6	166
< 1	78	106
**Echogenicity**		
Hypoechoic	29	258
Isoechoic or Mixed echoic	55	14
**Calcification**		
None	69	124
Microcalcification	10	120
Macrocalcification	5	28
Color Doppler character*		
Absent vascularity	32	137
Minimal vascularity	17	82
Moderate vascularity	20	35
Marked vascularity	15	18

*Color Doppler character was graded to be absent, minimal, moderate and marked. Absent vascularity: no vessel; minimal vascularity: point strip blood flow at 1–2 points, and the diameter of vessel was less than 1 mm; moderate vascularity: one vessel which diameter exceeded the radius of the lesion, or 3–4 point strip blood flow; marked vascularity: more than 4 vessels.

### Emax values of benign and malignant nodules for all lesions

The Emax values of the malignant nodules were significantly higher than those of the benign nodules across all lesions (*p* < 0.05). The median Emax values of the 272 malignant nodules and the 84 benign nodules were 51.85 kPa (36.40–71.58 kPa) and 24.00 kPa (17.35–34.85 kPa), respectively.

### Emax values of benign and malignant nodules in size groups

There were 220 nodules in Group A (44 benign and 176 malignant), 91 nodules in Group B (23 benign and 68 malignant), and 45 nodules in Group C (17 benign and 28 malignant). The median and interquartile ranges of each group’s Emax values for benign and malignant nodules are shown in Table 2.

**Table 2:** Emax values of benign and malignant thyroid nodules in each group.

Group	Emax of benign (kPa)	Emax of malignant (kPa)	*p*
A	22.6 (16.1,31.5)*	42.9 (31.0,58.3)#	< 0.001
B	24.4 (18.9,35.8)	64.0 (49.6,88.4)	< 0.001
C	36.1 (20.2,53.3)	87.1 (67.0,123.9)	< 0.001

Values are presented as the median (interquartile range). *indicates that the comparison of Emax values among the three groups had statistical significance for benign nodules (*p* = 0.03); # indicates that the comparison of Emax values among the three groups had statistical significance for malignant nodules (*p* < 0.001).

The Emax values of both benign and malignant nodules increased with nodule size according to the size groups. The differences in Emax values among the three groups were statistically significant for benign nodules (*p* = 0.03 for Groups A vs. B vs. C). The differences in Emax values among the three groups were also statistically significant for malignant nodules (*p* < 0.001 for Groups A vs. B vs. C) (Table 2).

The Emax values of malignant nodules were significantly higher than those of benign nodules in every size group (*p* < 0.001, *p* < 0.001, and *p* < 0.001 for Groups A, B, and C, respectively; Table 2).

### Comparison of diagnostic performance of Emax

**Figure 1:**
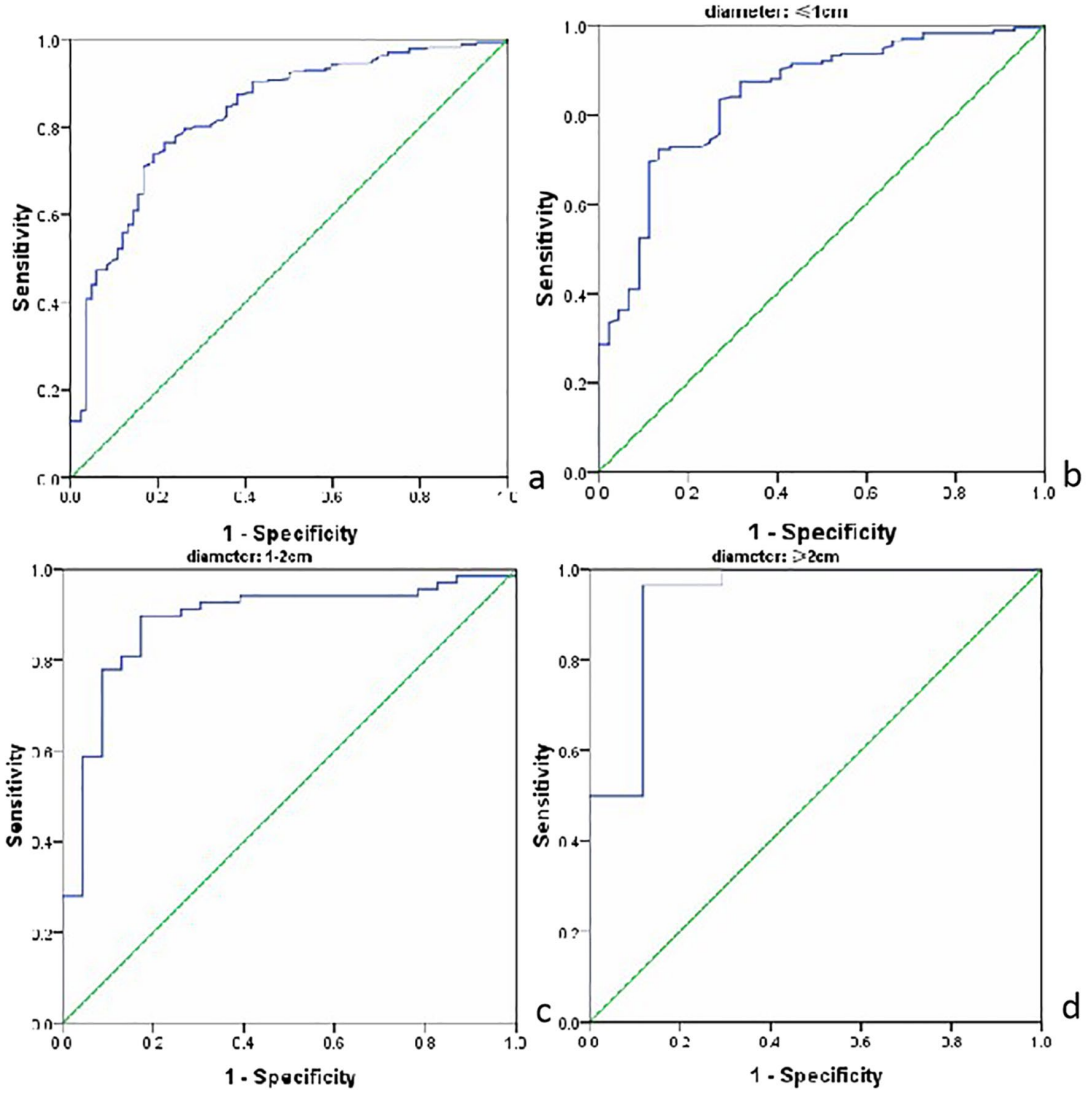
ROC curves to evaluate the efficacy of Emax in the diagnosis of malignant thyroid nodules before and after grouping. (**a**) ROC curve of entire dataset. (**b**) ROC curve of Group A. (**c**) ROC curve of Group B. (**d**) ROC curve of Group C. The figure was created by R software(version 3.4.4, url: https://www.R-project.org).

Taking the pathological results as the gold standard, we drew ROC curves to evaluate the efficacy of Emax in the diagnosis of malignant thyroid nodules with and without size-based grouping (Fig. 1). The AUC values of Groups A, B, and C increased to varying degrees compared with the corresponding values for all lesions, and the AUC value for Group C increased significantly. We used ROC curves to determine each group’s optimal cutoff points of Emax for the diagnosis of malignant thyroid nodules, the optimal point for the ROC curve was determined with Youden’s index. The diagnostic performance parameters are shown in Table 3.

**Table 3:** Diagnostic performance parameters of Emax values in each group.

Group	AUC	Cutoff point (kPa)	Sensitivity (%)	Specificity (%)	Youden's index	95% confidence interval
All lesions	0.830	36.2	76.5	78.4	0.549	0.780, 0.880
Group A	0.844	33.7*	72.2	86.4	0.586	0.781, 0.907
Group B	0.886	37.7**	89.7	82.6	0.723	0.805, 0.966
Group C	0.935	55.1^#^	96.4	88.2	0.846	0.853, 1.017

*Indicates the AUC of Group A compared with that of all lesions, z = 0.34, *p* > 0.05.

**Indicates the AUC of Group B compared with that of all lesions, z = 1.17, *p* > 0.05;# indicates the AUC of Group C compared with that of all lesions, z = 2.15, *p* < 0.05.

The AUC values of Groups A, B, and C for the diagnosis of malignant thyroid nodules by the Emax value were 0.844, 0.886, and 0.935, respectively. The AUC values of each group were higher than those of all lesions (0.830). There was a significant difference between the AUC value of Group C and that of all lesions (*p* < 0.05), but there were no significant differences between the AUC values of Groups A or B and that of all lesions (*p* > 0.05). The optimal cutoff points of Emax were 33.7 kPa, 37.7 kPa, and 55.1 kPa for Groups A, B, and C, respectively, and that for all lesions was 36.2 kPa. Compared with the value for all lesions, the optimal Emax values for Groups A, B, and C were lower, slightly higher, and higher, respectively. The sensitivity and specificity values for the diagnosis of malignant thyroid nodules were also compared between the size groups and all nodules. The specificity of Group A increased (86.4% vs. 78.4% for Group A vs. all lesions, respectively), while the specificity (82.6% and 88.2% vs. 78.4% for Groups B and C vs. all lesions, respectively) and sensitivity (89.7% and 96.4% vs. 76.5% for Groups B and C vs. all lesions, respectively) increased for both Groups B and C.

## Discussion

The value of SWE for differentiating benign from malignant thyroid nodules has been affirmed previously. In this study, the Emax values of malignant thyroid nodules were significantly higher than those of benign thyroid nodules, which is consistent with previously reported results^5,7,16,17^. However, the previously published threshold for diagnosing malignant nodules from benign nodules using the Emax value in SWE has varied considerably in this study (36.5 kPa–94.0 kPa)^4–13^. The optimal cutoff value of Emax obtained by ROC curve analysis in this study was 36.2 kPa. The optimal diagnostic threshold may vary with the size of the selected nodule. Previous researchers compared the Emax values of 230 nodules according to size and found that the Emax values of malignant nodules increased with nodule size, whereas the Emax values of benign nodules showed no significant differences between size groups^18^. Previous researchers have also observed that the Emax value increases with increasing nodule size, regardless of whether the nodules are benign or malignant^19–21^. In this study, 356 nodules were divided into three groups according to their maximum diameter: ≤ 1 cm, 1–2 cm, and ≥ 2 cm. In both benign and malignant nodules, an increased maximum diameter was associated with a corresponding increase in the median Emax value. The between-group differences in Emax values were statistically significant regardless of whether benign or malignant nodules were assessed. However, some researchers have opined that a nodule’s Emax value is not related to its size^6,15^. The reasons for these differences of opinion may be related to the studies’ sample sizes, the nodule size investigated in each study, and the proportions of benign and malignant nodules in each group.

In this study, the AUC of the Emax values of all nodules was 0.830. This value indicates that Emax was a valuable parameter in the diagnosis of benign and malignant thyroid nodules. When the optimal Emax cutoff value of 36.2 kPa was set, the sensitivity and specificity for the diagnosis of malignant thyroid nodules were 76.5% and 78.4%, respectively, for all nodules. After size grouping, the ROC curves of Groups A, B, and C were evaluated, and the optimal cutoff values were 33.7 kPa, 37.7 kPa, and 55.1 kPa, respectively. The specificity of Group A was improved, and the sensitivity and specificity of Groups B and C were significantly improved, compared with the values for all lesions. Meanwhile, the AUC values of Emax in Groups A, B, and C were higher than those for all lesions, with the greatest difference between Group C and all lesions. Thus, the results indicate that thyroid nodule size affects the diagnostic efficiency of SWE. Using different cutoff values for different sizes of thyroid nodules can improve the diagnostic efficiency of SWE.

The diagnostic sensitivity of the Emax value in Group A was lower than that in Groups B and Group C, which is consistent with results reported in the literature^14^. The sensitivity of the Emax value in Group A was lower than that of all lesions (72.2% vs. 76.5%). This can be attributed to the pathological basis of the malignant nodules in Group A: their diameter is ≤ 1 cm, and this type of nodule is also known as microcarcinoma. These nodules have the pathological characteristics of thyroid cancer, but their pathological changes are not obvious, and they have low levels of fiber content and gravel bodies^22^. This situation leads to the relatively low stiffness of these lesions, which has only slightly different values from the stiffness of benign nodules of the same size, thereby increasing the difficulty of SWE-based diagnosis. However, the Emax values in Groups B and C had high diagnostic sensitivity, consistent with the pathological development process of thyroid cancer, and these values were slightly higher than the results of previous research^18^, which may be related to the inclusion and exclusion criteria. This study excluded internal or peripheral nodules with coarse calcification, a decision that was based on previous studies^23^ showing that calcification can increase Emax. This is one of the main reasons for the high false-positive rate of SWE-based diagnosis of malignant nodules. Furthermore, coarse calcification is a US sign that indicates benign nodules, while microcalcification is a highly specific sign that predicts papillary thyroid carcinoma.

This study has some limitations. First, there may be selection bias because all the subjects were patients who underwent thyroid surgery. Thus, the number of malignant nodules was larger than that of benign nodules (272 and 84, respectively). Second, this study did not investigate diverse pathological types of lesions. The malignant nodules were all papillary carcinomas, and the benign nodules were mostly nodular goiter.

## Conclusions

SWE can provide accurate quantitative information about thyroid nodule stiffness, and nodule size affects the SWE imaging values and the efficiency of diagnosing malignant nodules. The size of the nodule affects the SWE imaging values and the efficiency of diagnosing malignant nodules. We suggest that different cutoff values be used for different sizes of lesions when SWE is used to diagnose thyroid nodules.

## Data Availability

The datasets analysed during the current study are available from the corresponding author on reasonable request.

## Abbreviations

AUC: The area under the ROC curve
Emax: The maximum value of Young's modulus
ROC: Receiver operating characteristic
SWE: Shear wave elastography
US: Ultrasonography
